# Crystal structures of the two epimers from the unusual thermal C6-epimerization of 5-oxo-1,2,3,5,5a,6,7,9b-octa­hydro-7,9a-ep­oxy­pyrrolo­[2,1-*a*]iso­indole-6-carb­oxy­lic acid, 5a(*RS*),6(*SR*),7(*RS*),9a(*SR*),9b(*SR*) and 5a(*RS*),6(*RS*),7(*RS*),9a(*SR*),9b(*SR*)

**DOI:** 10.1107/S2056989016014420

**Published:** 2016-09-16

**Authors:** Dmitry S. Poplevin, Fedor I. Zubkov, Pavel V. Dorovatovskii, Yan V. Zubavichus, Victor N. Khrustalev

**Affiliations:** a Organic Chemistry Department, Peoples’ Friendship University of Russia (RUDN University), 6 Miklukho-Maklay St., Moscow 117198, Russian Federation; b National Research Centre "Kurchatov Institute", 1 Acad. Kurchatov Sq., Moscow 123182, Russian Federation; c Inorganic Chemistry Department, Peoples’ Friendship University of Russia (RUDN University), 6 Miklukho-Maklay St., Moscow 117198, Russian Federation; d X-Ray Structural Centre, A.N. Nesmeyanov Institute of Organoelement Compounds, Russian Academy of Sciences, 28 Vavilov St., B–334, Moscow 119991, Russian Federation

**Keywords:** intra­molecular Diels–Alder furan (IMDAF) reaction, aza­heterocycles, epimerization, tautomerism, crystal structure

## Abstract

The isomeric title compounds, C_12_H_13_NO_4_ (I*a*) and C_12_H_13_NO_4_ (II*a*), the products of an usual thermal C6-epimerization of 5-oxo-1,2,3,5,5a,6,7,9b-octa­hydro-7,9a-ep­oxy­pyrrolo­[2,1-*a*]iso­indole-6-carb­oxy­lic acid, represent the two different diastereomers and have very similar mol­ecular geometries. The mol­ecules of both compounds comprise a fused tetra­cyclic system containing four five-membered rings (pyrrolidine, pyrrolidinone, di­hydro­furan and tetra­hydro­furan), all of which adopt the usual envelope conformations. The dihedral angle between the basal planes of the pyrrolidine and pyrrolidinone rings are 14.3 (2) and 16.50 (11)°, respectively, for (I*a*) and (II*a*). The nitro­gen atom has a slightly pyramidalized geometry [bond-angle sum = 355.9 and 355.3°, for (I*a*) and (II*a*)], respectively. In the crystal of (I*a*), mol­ecules form zigzag-like hydrogen-bonded chains along [010] through strong O—H⋯O hydrogen bonds and are further linked by weak C—H⋯O hydrogen bonds into complex two-tier layers parallel to (100). Unlike (I*a*), the crystal of (II*a*) contains centrosymmetric cyclic hydrogen-bonded dimers [graph set *R* ^2^_2_(14)], formed through strong O—H⋯O hydrogen bonds and are further linked by weak C—H⋯O hydrogen bonds into ribbons extending across [101].

## Abstract 2

The two epimers from the unusual thermal C6-epimerization of 5-oxo-1,2,3,5,5a,6,7,9b-octa­hydro-7,9a-ep­oxy­pyrrolo­[2,1-*a*]iso­indole-6-carb­oxy­lic acid, 5a(*RS*),6(*SR*),7(*RS*),9a(*SR*),9b(*SR*) and 5a(*RS*),6(*RS*),7(*RS*),9a(*SR*),9b(*SR*) have similar geometries but differ in their hydrogen-bonded crystal-packing modes

## Chemical context   

The intra­molecular Diels–Alder furan (IMDAF) reaction between α,β-unsaturated acid anhydrides and hydrogenated heterocycles, containing a furfuryl­amine moiety, has been studied for a long time (see, for example, Parker & Adamchuk, 1978 ▸; Blokzijl *et al.*, 1991 ▸; Varlamov *et al.*, 2006 ▸; Groenendaal *et al.*, 2008 ▸; Nakamura *et al.*, 2011 ▸; Zubkov *et al.*, 2011 ▸, 2012 ▸, 2014 ▸; Toze *et al.*, 2015 ▸) and used for diastereospecific synthesis of diverse fused-ring systems. It is arguable that the pathway with a simultaneous controlled formation of four or five new stereogenic centers is the best approach to ep­oxy­iso­indoles and affords target adducts under mild conditions with satisfactory yields. However, the simplest 2-furyl aza­heterocycles (azetidine, pyrrolidine, piperidine, perhydro­azepine) have not yet been studied in this reaction. One of the goals of our work is to fill the gap. Here we report on the utilization of 2-furyl pyrrolidine as an initial reagent in the IMDAF reaction.
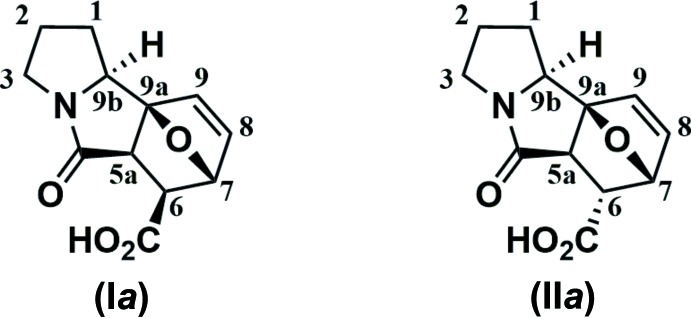


**Figure 1:**
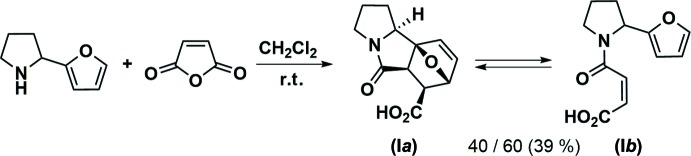
Reaction of 2-furyl pyrrolidine and maleic anhydride at room temperature.

**Figure 2:**
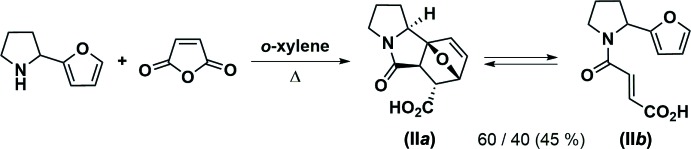
Reaction of 2-furyl pyrrolidine and maleic anhydride at 413 K.

The inter­action between 2-furyl pyrrolidine and maleic anhydride at room temperature leads to the mixture of cyclic (I*a*) and open-chain (I*b*) tautomers, the crystallization of which results in the cyclic form (I*a*) only (Fig. 1 ▸). In contrast, the same reaction at 413 K leads to the maleic amide fragment isomerization and affords a mixture of the adduct (II*a*) and the amide (II*b*) (Fig. 2 ▸). Similarly, the mixture crystallization gives rise the cyclic tautomer (II*a*) only. The crystal structures of both (I*a*) and (II*a*) using synchrotron X-ray diffraction data have been determined and are reported herein.

## Structural commentary   

**Figure 3:**
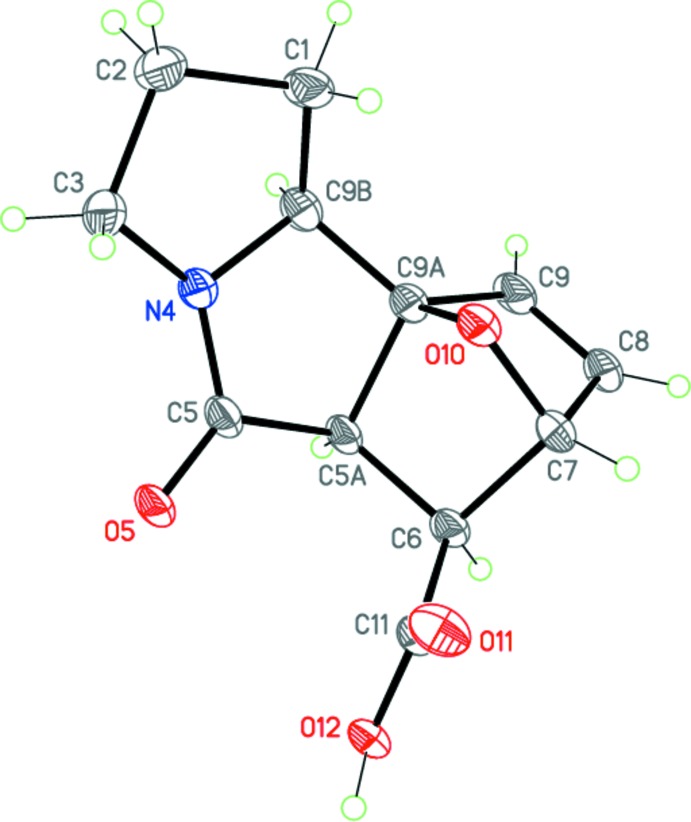
Mol­ecular structure and atom-numbering scheme for epimer (I*a*). Displacement ellipsoids are drawn at the 50% probability level. H atoms are presented as small spheres of arbitrary radius.

Compounds (I*a*) and (II*a*) represent two different diastereo­mers of 5-oxo-1,2,3,5,5a,6,7,9b-octa­hydro-7,9a-ep­oxy­pyrrolo[2,1-*a*]iso­indole-6-carb­oxy­lic acid and have very similar mol­ecular geometries (Figs. 3 ▸, 4 ▸). The mol­ecules of (I*a*) and (II*a*) each comprise a fused tetra­cyclic system containing four five-membered rings (pyrrolidine, pyrrolidinone, di­hydro­furan and tetra­hydro­furan), all of which adopt the usual envelope conformations. The dihedral angles between the basal planes of the pyrrolidine and pyrrolidinone rings are 14.3 (2) and 16.50 (11)°, respectively, for (I*a*) and (II*a*). The nitro­gen N4 atom has a slightly pyramidalized geometry [sum of the bond angles = 355.9 and 355.3°, respectively, for (I*a*) and (II*a*)]. The bond lengths and angles in both epimers are in good agreement with those observed in a related structure (Lu *et al.*, 2013 ▸).

The mol­ecules possess five asymmetric centers at the C5, C6, C7, C9a and C9b carbon atoms. The crystals of (I*a*) and (II*a*) are racemic and consist of enanti­omeric pairs with the following relative configurations of the centers: 5a(*RS*),6(*SR*),7(*RS*),9a(*SR*),9b(*SR*) and 5a(*RS*),6(*RS*),7(*RS*),9a(*SR*),9b(*SR*)

## Supra­molecular features   

**Figure 5:**
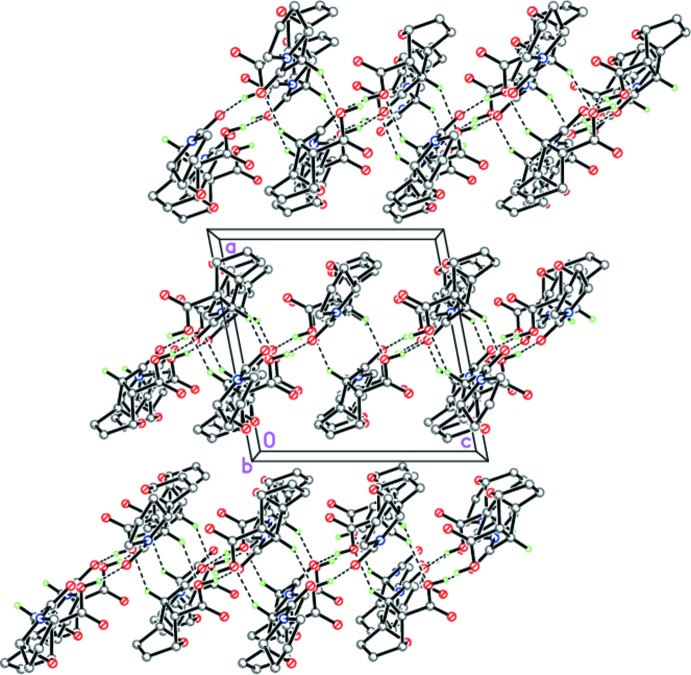
Crystal structure of (I*a*) showing the two-tier layers parallel to (100). Dashed lines indicate the inter­molecular O—H⋯O and C—H⋯O hydrogen bonds.

Although the similarity of the mol­ecular geometries might lead to similar packing motifs, this is not found in the case of (I*a*) and (II*a*). The inter­molecular inter­actions, namely strong O—H⋯O and weak C—H⋯O hydrogen bonding, combined in a different way, give rise to different packing networks. In the crystal of (I*a*), mol­ecules form zigzag-like hydrogen-bonded chains extending along [010] through strong O12—H12⋯O5^i^ hydrogen bonds, which are further linked by weak C5*A*—H5*A*⋯O12^ii^ hydrogen bonds into complex two-tier layers lying parallel to (100) (Table 1 ▸, Fig. 5 ▸).

**Figure 6:**
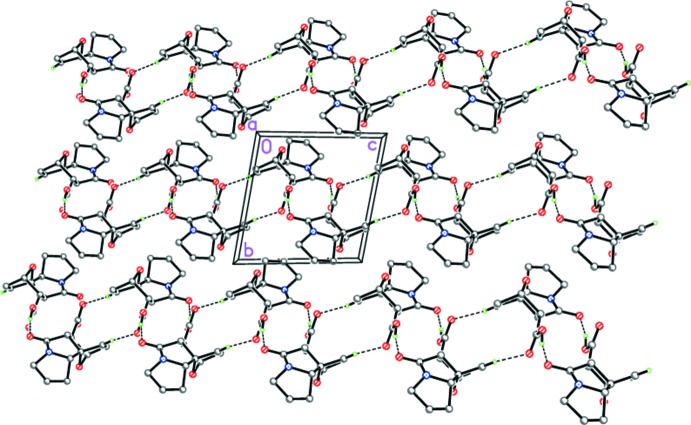
The hydrogen-bonded chains of (II*a*). Dashed lines indicate the inter­molecular O—H⋯O and C—H⋯O hydrogen bonds.

However, unlike (I*a*), the crystal of (II*a*) contains centrosymmetric hydrogen-bonded cyclic dimers [graph set 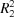
(14), formed through two strong O12—H12⋯O5^i^ hydrogen bonds (Table 2 ▸, Fig. 6 ▸). The dimers are further linked by weak C9—H9⋯O11^ii^ hydrogen bonds into ribbons extending across [101] (Table 2 ▸, Figs. 6 ▸ and 7 ▸).

## Synthesis and crystallization   

The initial 2-furyl pyrrolidine was synthesized according to the procedure described previously (Acher *et al.*, 1981 ▸; Shono *et al.*, 1981 ▸; Nikolic & Beak, 1997 ▸).

**Synthesis of (I** ***a*** **).** A mixture of the initial 2-furyl pyrrolidine (0.30 g, 2.2 mmol) and maleic anhydride (0.23 g, 2.3 mmol) in di­chloro­methane (6 mL) was stirred for 5 h at r.t. [monitoring by TLC until disappearance of the starting compound spot, eluent–EtOAc: hexane (1:3), Sorbfil]. On completion of the reaction, the solvent was evaporated. The isomer (I*a*) was isolated as fine needles by slow recrystallization of the residue from a mixture of EtOAc–EtOH. Yield 39%: m.p. = 413–414 K. IR (KBr), ν (cm^−1^): 1734, 1654. ^1^H NMR (CDCl_3_, 400 MHz, 300 K): δ = 1.98–1.69 (*m*, 4H, H1a, H1b, H2a, H2b), 2.42 (*d*, 1H, H6, *J*_6,5a_ = 9.1), 2.94–2.88 (*m*, 2H, H3a, H3b), 3.10 (*d*, 1H, H5a, *J*_5a,6_ = 9.1), 4.41 (*t*, 1H, H9b, *J*_9b,1a_ = 7.5, *J*_9b,1 b_ = 7.5), 4.97 (*d*, 1H, H7, *J*_7,8_ = 1.6), 6.44 (*dd*, 1H, H8, *J*_8,9_ = 5.5, *J*_8,7_ = 1.6), 6.54 (*d*, 1H, H9, *J*_9,8_ = 5.5). ^13^C NMR (CDCl_3_, 100 MHz, 300 K): δ = 23.5, 26.2, 41.9 (C1, C2, C3), 46.9, 53.8 (C6, C5a), 60.0 (C9b), 80.5 (C7), 93.5 (C9a), 133.9 (C9), 137.1 (C8), 171.7, 173.2 (NCO, COOH). EI–MS (70 eV), *m*/*z* (rel. intensity): 235 (22), 217 (91), 137 (41), 136 (100), 108 (39), 80 (45), 70 (32), 54 (38), 45 (29), 42 (25).

**Synthesis of (II** ***a*** **).** A mixture of the initial 2-furyl pyrrolidine (0.3 g, 2.2 mmol) and maleic anhydride (0.23 g, 2.3 mmol) in *o*-xylene (6 mL) was heated at reflux for 3 h. At the end of the reaction, the solvent was evaporated. The isomer (II*a*) was isolated as fine needles by slow recrystallization of the residue from a mixture of EtOAc–EtOH. Yield: 0.33 45%; m.p. = 414–416 K. IR (KBr), ν (cm^−1^): 1738, 1658. ^1^H NMR (CDCl_3_, 400 MHz, 300 K): δ = 1.82–1.64 (*m*, 4H, H1a, H1b, H2a, H2b), 3.02 (*d*, 1H, H5a, *J*_5a,6_ = 3.4), 3.17 (*dd*, 1H, H6, *J*_6,5a_ = 3.4, *J*_6,5a_ = 3.4), 3.36–3.32 (*m*, 2H, H3a, H3b), 4.52 (*t*, 1H, H9b, *J*_9b,1a_ = 7.6, *J*_9b,1b_ = 7.6), 5.20 (*dd*, 1H, H7, *J*_7,8_ = 1.6, *J*_7,6_ = 4.8), 6.34 (dd, 1H, H8, *J*_8,9_ = 5.8, *J*_8,7_ = 1.6), 6.66 (*d*, 1H, H9, *J*_9,8_ = 5.8). ^13^C NMR (CDCl_3_, 100 MHz, 300 K): δ = 23.5, 26.2, 42.1 (C1, C2, C3), 47.0, 55.1 (C6, C5a), 61.0 (C9b), 79.2 (C7), 93.5 (C9a), 133.9 (C8), 135.2 (C9), 171.7, 173.2 (NCO, COOH). EI–MS (70 eV), *m*/*z* (rel. intensity): 235 (22), 217 (91), 137 (41), 136 (100), 108 (39), 80 (45), 70 (32), 54 (38), 45 (29), 42 (25).

## Refinement   

Crystal data, data collection and refinement details are summarized in Table 3 ▸. X-ray diffraction studies were carried out on the ‘Belok’ beamline (λ = 0.96990 Å) of the National Research Center "Kurchatov Institute" (Moscow, Russian Federation) using a MAR CCD detector.

The hydrogen atoms of the hydroxyl groups were localized in the difference-Fourier maps and refined in an isotropic approximation with fixed displacement parameters [*U*_iso_(H) = 1.5*U*_eq_(O)] [for (I*a*)] or included in the refinement with fixed positional (riding model) and isotropic displacement parameters [*U*_iso_(H) = 1.5*U*_eq_(O)] [for (II*a*)]. Other hydrogen atoms were placed in calculated positions with C—H = 0.95–1.00 Å and refined in the riding model with fixed isotropic displacement parameters [*U*_iso_(H) = 1.2*U*_eq_(C)].

The insufficient data completeness of 94.1% in the case of (II*a*) is the result of the low (triclinic) crystal symmetry, making it very difficult to obtain good data completeness using the φ scan mode only (‘Belok’ beamline limitation), even though we have used the two different crystal orientations.

## Supplementary Material

Crystal structure: contains datablock(s) global, Ia, IIa. DOI: 10.1107/S2056989016014420/zs2368sup1.cif

Structure factors: contains datablock(s) Ia. DOI: 10.1107/S2056989016014420/zs2368Iasup2.hkl

Structure factors: contains datablock(s) IIa. DOI: 10.1107/S2056989016014420/zs2368IIasup3.hkl

CCDC references: 1503860, 1503859

Additional supporting information:  crystallographic information; 3D view; checkCIF report

## Figures and Tables

**Figure 4:**
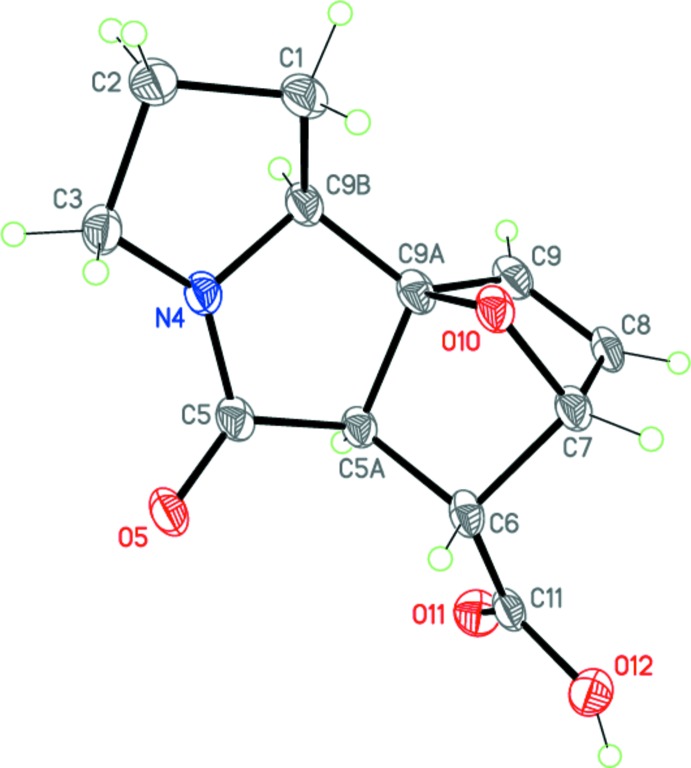
Mol­ecular structure and atom-numbering scheme for epimer (II*a*). Displacement ellipsoids are drawn at the 50% probability level. H atoms are presented as small spheres of arbitrary radius.

**Figure 7:**
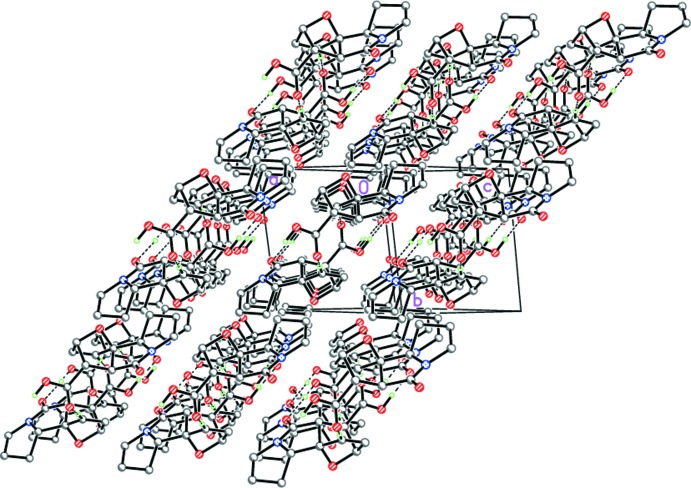
Crystal structure of (II*a*) along [101]. Dashed lines indicate the inter­molecular O—H⋯O and C—H⋯O hydrogen bonds.

**Table 1:** Hydrogen-bond geometry (Å, °) for (I*a*)

*D*—H⋯*A*	*D*—H	H⋯*A*	*D*⋯*A*	*D*—H⋯*A*
O12—H12⋯O5^i^	0.90 (3)	1.75 (3)	2.613 (2)	157 (3)
C5*A*—H5*A*⋯O12^ii^	1.00	2.51	3.234 (3)	129

Symmetry codes: (i) 
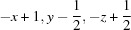
; (ii) 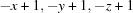.

**Table 2:** Hydrogen-bond geometry (Å, °) for (II*a*)

*D*—H⋯*A*	*D*—H	H⋯*A*	*D*⋯*A*	*D*—H⋯*A*
O12—H12⋯O5^i^	0.92	1.70	2.607 (2)	165
C9—H9⋯O11^ii^	0.95	2.42	3.362 (3)	172

Symmetry codes: (i) 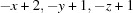; (ii) 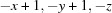.

**Table 3:** Experimental details

	(I*a*)	(II*a*)
Crystal data		
Chemical formula	C_12_H_13_NO_4_	C_12_H_13_NO_4_
*M*_r_	235.23	235.23
Crystal system, space group	Monoclinic, *P*2_1_/*c*	Triclinic, *P* 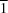
Temperature (K)	100	100
*a*, *b*, *c* (Å)	11.045 (2), 9.2023 (18), 11.062 (2)	8.4700 (17), 8.5100 (17), 8.5900 (17)
α, β, γ (°)	90, 100.91 (3), 90	94.04 (3), 111.12 (3), 105.17 (3)
*V* (Å^3^)	1104.1 (4)	548.0 (2)
*Z*	4	2
Radiation type	Synchrotron, λ = 0.96990 Å	Synchrotron, λ = 0.96990 Å
μ (mm^−1^)	0.23	0.23
Crystal size (mm)	0.20 × 0.15 × 0.15	0.15 × 0.10 × 0.10

Data collection		
Diffractometer	MAR CCD	MAR CCD
Absorption correction	Multi-scan (*SCALA*; Evans, 2006 ▸)	Multi-scan (*SCALA*; Evans, 2006 ▸)
*T*_min_, *T*_max_	0.950, 0.960	0.960, 0.969
No. of measured, independent and observed [*I* > 2σ(*I*)] reflections	12183, 2329, 1864	7090, 2104, 1402
*R*_int_	0.085	0.061
(sin θ/λ)_max_ (Å^−1^)	0.641	0.637

Refinement		
*R*[*F* ^2^ > 2σ(*F* ^2^)], *wR*(*F* ^2^), *S*	0.072, 0.189, 1.02	0.099, 0.240, 0.93
No. of reflections	2329	2104
No. of parameters	158	155
H-atom treatment	H atoms treated by a mixture of independent and constrained refinement	H-atom parameters constrained
Δρ_max_, Δρ_min_ (e Å^−3^)	0.43, −0.43	0.45, −0.36

Computer programs: *Automar* (MarXperts, 2015 ▸), *i* *MOSFLM* (Battye *et al.*, 2011 ▸), *SHELXS97* and *SHELXTL* (Sheldrick, 2008 ▸) and *SHELXL2014* (Sheldrick, 2015 ▸).

## supplementary crystallographic information

### (Ia) 5a(RS),6(SR),7(RS),9a(SR),9b(SR)-5-Oxo-1,2,3,5,5a,6,7,9b-octahydro-7,9a-epoxypyrrolo[2,1-a]isoindole-6-carboxylic acid Crystal data

**Table:**

C_12_H_13_NO_4_	*D*_x_ = 1.415 Mg m^−^^3^
*M**_r_* = 235.23	Melting point = 413–414 K
Monoclinic, *P*2_1_/*c*	Synchrotron radiation, λ = 0.96990 Å
*a* = 11.045 (2) Å	Cell parameters from 600 reflections
*b* = 9.2023 (18) Å	θ = 4.5–38.0°
*c* = 11.062 (2) Å	µ = 0.23 mm^−^^1^
β = 100.91 (3)°	*T* = 100 K
*V* = 1104.1 (4) Å^3^	Prism, colourless
*Z* = 4	0.20 × 0.15 × 0.15 mm
*F*(000) = 496	

### (Ia) 5a(RS),6(SR),7(RS),9a(SR),9b(SR)-5-Oxo-1,2,3,5,5a,6,7,9b-octahydro-7,9a-epoxypyrrolo[2,1-a]isoindole-6-carboxylic acid Data collection

**Table:**

MAR CCD diffractometer	1864 reflections with *I* > 2σ(*I*)
φ scan	*R*_int_ = 0.085
Absorption correction: multi-scan (*SCALA*; Evans, 2006)	θ_max_ = 38.5°, θ_min_ = 4.5°
*T*_min_ = 0.950, *T*_max_ = 0.960	*h* = −13→14
12183 measured reflections	*k* = −11→10
2329 independent reflections	*l* = −13→13

### (Ia) 5a(RS),6(SR),7(RS),9a(SR),9b(SR)-5-Oxo-1,2,3,5,5a,6,7,9b-octahydro-7,9a-epoxypyrrolo[2,1-a]isoindole-6-carboxylic acid Refinement

**Table:**

Refinement on *F*^2^	Secondary atom site location: difference Fourier map
Least-squares matrix: full	Hydrogen site location: mixed
*R*[*F*^2^ > 2σ(*F*^2^)] = 0.072	H atoms treated by a mixture of independent and constrained refinement
*wR*(*F*^2^) = 0.189	*w* = 1/[σ^2^(*F*_o_^2^) + (0.08*P*)^2^ + 1.*P*] where *P* = (*F*_o_^2^ + 2*F*_c_^2^)/3
*S* = 1.02	(Δ/σ)_max_ < 0.001
2329 reflections	Δρ_max_ = 0.43 e Å^−^^3^
158 parameters	Δρ_min_ = −0.43 e Å^−^^3^
0 restraints	Extinction correction: SHELXL-2014/7 (Sheldrick, 2015), Fc^*^=kFc[1+0.001xFc^2^λ^3^/sin(2θ)]^-1/4^
Primary atom site location: difference Fourier map	Extinction coefficient: 0.104 (9)

### (Ia) 5a(RS),6(SR),7(RS),9a(SR),9b(SR)-5-Oxo-1,2,3,5,5a,6,7,9b-octahydro-7,9a-epoxypyrrolo[2,1-a]isoindole-6-carboxylic acid Special details

**Table:**

Geometry. All esds (except the esd in the dihedral angle between two l.s. planes) are estimated using the full covariance matrix. The cell esds are taken into account individually in the estimation of esds in distances, angles and torsion angles; correlations between esds in cell parameters are only used when they are defined by crystal symmetry. An approximate (isotropic) treatment of cell esds is used for estimating esds involving l.s. planes.

### (Ia) 5a(RS),6(SR),7(RS),9a(SR),9b(SR)-5-Oxo-1,2,3,5,5a,6,7,9b-octahydro-7,9a-epoxypyrrolo[2,1-a]isoindole-6-carboxylic acid Fractional atomic coordinates and isotropic or equivalent isotropic displacement parameters (Å^2^)

**Table:**

	*x*	*y*	*z*	*U*_iso_*/*U*_eq_	
C1	0.8345 (2)	0.9330 (3)	0.5467 (2)	0.0330 (6)	
H1A	0.8888	0.8929	0.4933	0.040*	
H1B	0.8859	0.9780	0.6202	0.040*	
C2	0.7415 (2)	1.0430 (3)	0.4762 (2)	0.0349 (6)	
H2A	0.7799	1.1002	0.4177	0.042*	
H2B	0.7123	1.1106	0.5341	0.042*	
C3	0.6333 (2)	0.9507 (3)	0.4064 (2)	0.0312 (6)	
H3A	0.5531	0.9989	0.4059	0.037*	
H3B	0.6426	0.9320	0.3205	0.037*	
N4	0.64418 (17)	0.8166 (2)	0.47832 (17)	0.0272 (5)	
C5	0.60154 (19)	0.6839 (2)	0.4418 (2)	0.0253 (5)	
O5	0.52224 (14)	0.65677 (18)	0.34888 (14)	0.0280 (5)	
C5A	0.66529 (19)	0.5736 (2)	0.53586 (19)	0.0236 (5)	
H5A	0.6166	0.5553	0.6019	0.028*	
C6	0.7153 (2)	0.4306 (2)	0.4908 (2)	0.0254 (5)	
H6	0.7007	0.3500	0.5470	0.030*	
C7	0.8561 (2)	0.4655 (3)	0.5143 (2)	0.0296 (6)	
H7	0.9050	0.4068	0.4645	0.035*	
C8	0.9021 (2)	0.4591 (3)	0.6528 (2)	0.0330 (6)	
H8	0.9504	0.3847	0.6980	0.040*	
C9	0.8604 (2)	0.5799 (3)	0.6976 (2)	0.0302 (6)	
H9	0.8737	0.6103	0.7812	0.036*	
C9A	0.7874 (2)	0.6583 (2)	0.5868 (2)	0.0251 (5)	
C9B	0.7500 (2)	0.8164 (3)	0.5827 (2)	0.0291 (6)	
H9B	0.7224	0.8442	0.6606	0.035*	
O10	0.85603 (14)	0.62038 (18)	0.49215 (14)	0.0276 (4)	
C11	0.6649 (2)	0.3852 (2)	0.3585 (2)	0.0251 (5)	
O11	0.71540 (16)	0.4123 (2)	0.27256 (16)	0.0374 (5)	
O12	0.56401 (14)	0.30469 (18)	0.35042 (15)	0.0265 (4)	
H12	0.539 (3)	0.275 (3)	0.272 (3)	0.040*	

### (Ia) 5a(RS),6(SR),7(RS),9a(SR),9b(SR)-5-Oxo-1,2,3,5,5a,6,7,9b-octahydro-7,9a-epoxypyrrolo[2,1-a]isoindole-6-carboxylic acid Atomic displacement parameters (Å^2^)

**Table:**

	*U*^11^	*U*^22^	*U*^33^	*U*^12^	*U*^13^	*U*^23^
C1	0.0350 (13)	0.0293 (13)	0.0289 (12)	−0.0063 (9)	−0.0086 (10)	−0.0015 (9)
C2	0.0433 (15)	0.0252 (13)	0.0318 (14)	−0.0031 (10)	−0.0039 (11)	0.0009 (9)
C3	0.0333 (13)	0.0261 (13)	0.0304 (13)	0.0025 (9)	−0.0033 (10)	0.0043 (9)
N4	0.0257 (10)	0.0236 (11)	0.0278 (10)	0.0004 (7)	−0.0064 (8)	0.0017 (7)
C5	0.0192 (10)	0.0290 (12)	0.0246 (11)	−0.0003 (8)	−0.0036 (8)	0.0016 (8)
O5	0.0233 (8)	0.0293 (9)	0.0259 (9)	−0.0010 (6)	−0.0098 (6)	0.0025 (6)
C5A	0.0190 (10)	0.0270 (12)	0.0214 (11)	−0.0001 (8)	−0.0052 (8)	0.0018 (8)
C6	0.0229 (11)	0.0254 (12)	0.0235 (11)	−0.0011 (8)	−0.0066 (8)	0.0011 (8)
C7	0.0215 (11)	0.0309 (13)	0.0314 (13)	0.0017 (9)	−0.0075 (9)	−0.0039 (9)
C8	0.0252 (11)	0.0324 (13)	0.0334 (14)	0.0030 (9)	−0.0148 (9)	0.0008 (9)
C9	0.0268 (12)	0.0341 (14)	0.0237 (12)	−0.0021 (9)	−0.0102 (9)	0.0011 (9)
C9A	0.0231 (11)	0.0260 (12)	0.0226 (11)	−0.0001 (8)	−0.0045 (8)	−0.0008 (8)
C9B	0.0269 (12)	0.0323 (14)	0.0236 (11)	0.0001 (9)	−0.0066 (9)	−0.0003 (9)
O10	0.0222 (8)	0.0303 (9)	0.0276 (9)	−0.0023 (6)	−0.0023 (6)	−0.0015 (7)
C11	0.0221 (11)	0.0246 (12)	0.0254 (12)	0.0003 (8)	−0.0036 (8)	0.0000 (8)
O11	0.0352 (10)	0.0471 (12)	0.0276 (10)	−0.0085 (8)	0.0004 (7)	−0.0016 (7)
O12	0.0205 (8)	0.0301 (9)	0.0251 (9)	−0.0027 (6)	−0.0053 (6)	−0.0030 (6)

### (Ia) 5a(RS),6(SR),7(RS),9a(SR),9b(SR)-5-Oxo-1,2,3,5,5a,6,7,9b-octahydro-7,9a-epoxypyrrolo[2,1-a]isoindole-6-carboxylic acid Geometric parameters (Å, º)

**Table:**

C1—C9B	1.524 (4)	C6—C11	1.522 (3)
C1—C2	1.544 (3)	C6—C7	1.561 (3)
C1—H1A	0.9900	C6—H6	1.0000
C1—H1B	0.9900	C7—O10	1.446 (3)
C2—C3	1.548 (3)	C7—C8	1.522 (3)
C2—H2A	0.9900	C7—H7	1.0000
C2—H2B	0.9900	C8—C9	1.334 (4)
C3—N4	1.461 (3)	C8—H8	0.9500
C3—H3A	0.9900	C9—C9A	1.516 (3)
C3—H3B	0.9900	C9—H9	0.9500
N4—C5	1.343 (3)	C9A—O10	1.447 (3)
N4—C9B	1.480 (3)	C9A—C9B	1.511 (3)
C5—O5	1.243 (3)	C9B—H9B	1.0000
C5—C5A	1.527 (3)	C11—O11	1.216 (3)
C5A—C6	1.546 (3)	C11—O12	1.327 (3)
C5A—C9A	1.568 (3)	O12—H12	0.90 (3)
C5A—H5A	1.0000		
			
C9B—C1—C2	102.2 (2)	C11—C6—H6	108.8
C9B—C1—H1A	111.3	C5A—C6—H6	108.8
C2—C1—H1A	111.3	C7—C6—H6	108.8
C9B—C1—H1B	111.3	O10—C7—C8	101.44 (18)
C2—C1—H1B	111.3	O10—C7—C6	101.91 (17)
H1A—C1—H1B	109.2	C8—C7—C6	107.0 (2)
C1—C2—C3	105.63 (19)	O10—C7—H7	115.0
C1—C2—H2A	110.6	C8—C7—H7	115.0
C3—C2—H2A	110.6	C6—C7—H7	115.0
C1—C2—H2B	110.6	C9—C8—C7	105.6 (2)
C3—C2—H2B	110.6	C9—C8—H8	127.2
H2A—C2—H2B	108.7	C7—C8—H8	127.2
N4—C3—C2	102.45 (18)	C8—C9—C9A	105.3 (2)
N4—C3—H3A	111.3	C8—C9—H9	127.3
C2—C3—H3A	111.3	C9A—C9—H9	127.3
N4—C3—H3B	111.3	O10—C9A—C9B	112.83 (19)
C2—C3—H3B	111.3	O10—C9A—C9	101.49 (18)
H3A—C3—H3B	109.2	C9B—C9A—C9	125.67 (19)
C5—N4—C3	128.07 (19)	O10—C9A—C5A	98.78 (16)
C5—N4—C9B	114.43 (18)	C9B—C9A—C5A	104.85 (17)
C3—N4—C9B	113.42 (18)	C9—C9A—C5A	110.12 (18)
O5—C5—N4	125.7 (2)	N4—C9B—C9A	101.29 (17)
O5—C5—C5A	126.3 (2)	N4—C9B—C1	103.16 (18)
N4—C5—C5A	108.04 (17)	C9A—C9B—C1	120.6 (2)
C5—C5A—C6	119.56 (18)	N4—C9B—H9B	110.3
C5—C5A—C9A	99.73 (17)	C9A—C9B—H9B	110.3
C6—C5A—C9A	101.74 (17)	C1—C9B—H9B	110.3
C5—C5A—H5A	111.5	C7—O10—C9A	95.65 (17)
C6—C5A—H5A	111.5	O11—C11—O12	124.4 (2)
C9A—C5A—H5A	111.5	O11—C11—C6	123.9 (2)
C11—C6—C5A	117.10 (17)	O12—C11—C6	111.56 (19)
C11—C6—C7	112.8 (2)	C11—O12—H12	109.4 (19)
C5A—C6—C7	100.16 (17)		
			
C9B—C1—C2—C3	−35.5 (3)	C6—C5A—C9A—O10	40.32 (19)
C1—C2—C3—N4	23.1 (3)	C5—C5A—C9A—C9B	33.7 (2)
C2—C3—N4—C5	−157.3 (2)	C6—C5A—C9A—C9B	156.88 (18)
C2—C3—N4—C9B	−1.6 (3)	C5—C5A—C9A—C9	171.42 (19)
C3—N4—C5—O5	−16.7 (4)	C6—C5A—C9A—C9	−65.4 (2)
C9B—N4—C5—O5	−172.2 (2)	C5—N4—C9B—C9A	13.2 (3)
C3—N4—C5—C5A	164.6 (2)	C3—N4—C9B—C9A	−146.0 (2)
C9B—N4—C5—C5A	9.0 (3)	C5—N4—C9B—C1	138.6 (2)
O5—C5—C5A—C6	45.6 (3)	C3—N4—C9B—C1	−20.6 (3)
N4—C5—C5A—C6	−135.7 (2)	O10—C9A—C9B—N4	77.5 (2)
O5—C5—C5A—C9A	155.1 (2)	C9—C9A—C9B—N4	−157.9 (2)
N4—C5—C5A—C9A	−26.1 (2)	C5A—C9A—C9B—N4	−29.0 (2)
C5—C5A—C6—C11	−18.4 (3)	O10—C9A—C9B—C1	−35.3 (3)
C9A—C5A—C6—C11	−126.8 (2)	C9—C9A—C9B—C1	89.3 (3)
C5—C5A—C6—C7	103.9 (2)	C5A—C9A—C9B—C1	−141.8 (2)
C9A—C5A—C6—C7	−4.5 (2)	C2—C1—C9B—N4	33.3 (2)
C11—C6—C7—O10	92.5 (2)	C2—C1—C9B—C9A	145.2 (2)
C5A—C6—C7—O10	−32.8 (2)	C8—C7—O10—C9A	−50.66 (19)
C11—C6—C7—C8	−161.48 (19)	C6—C7—O10—C9A	59.66 (18)
C5A—C6—C7—C8	73.2 (2)	C9B—C9A—O10—C7	−171.63 (16)
O10—C7—C8—C9	32.0 (3)	C9—C9A—O10—C7	51.38 (18)
C6—C7—C8—C9	−74.4 (2)	C5A—C9A—O10—C7	−61.36 (16)
C7—C8—C9—C9A	0.9 (3)	C5A—C6—C11—O11	95.3 (3)
C8—C9—C9A—O10	−33.6 (2)	C7—C6—C11—O11	−20.2 (3)
C8—C9—C9A—C9B	−162.9 (2)	C5A—C6—C11—O12	−88.8 (2)
C8—C9—C9A—C5A	70.3 (3)	C7—C6—C11—O12	155.70 (19)
C5—C5A—C9A—O10	−82.84 (18)		

### (Ia) 5a(RS),6(SR),7(RS),9a(SR),9b(SR)-5-Oxo-1,2,3,5,5a,6,7,9b-octahydro-7,9a-epoxypyrrolo[2,1-a]isoindole-6-carboxylic acid Hydrogen-bond geometry (Å, º)

**Table:**

*D*—H···*A*	*D*—H	H···*A*	*D*···*A*	*D*—H···*A*
O12—H12···O5^i^	0.90 (3)	1.75 (3)	2.613 (2)	157 (3)
C5*A*—H5*A*···O12^ii^	1.00	2.51	3.234 (3)	129

Symmetry codes: (i) −*x*+1, *y*−1/2, −*z*+1/2; (ii) −*x*+1, −*y*+1, −*z*+1.

### (IIa) 5a(RS),6(RS),7(RS),9a(SR),9b(SR)-5-Oxo-1,2,3,5,5a,6,7,9b-octahydro-7,9a-epoxypyrrolo[2,1-a]isoindole-6-carboxylic acid Crystal data

**Table:**

C_12_H_13_NO_4_	*F*(000) = 248
*M**_r_* = 235.23	*D*_x_ = 1.426 Mg m^−^^3^
Triclinic, *P*1	Melting point = 414–416 K
*a* = 8.4700 (17) Å	Synchrotron radiation, λ = 0.96990 Å
*b* = 8.5100 (17) Å	Cell parameters from 500 reflections
*c* = 8.5900 (17) Å	θ = 3.6–36.0°
α = 94.04 (3)°	µ = 0.23 mm^−^^1^
β = 111.12 (3)°	*T* = 100 K
γ = 105.17 (3)°	Prism, colourless
*V* = 548.0 (2) Å^3^	0.15 × 0.10 × 0.10 mm
*Z* = 2	

### (IIa) 5a(RS),6(RS),7(RS),9a(SR),9b(SR)-5-Oxo-1,2,3,5,5a,6,7,9b-octahydro-7,9a-epoxypyrrolo[2,1-a]isoindole-6-carboxylic acid Data collection

**Table:**

MAR CCD diffractometer	1402 reflections with *I* > 2σ(*I*)
φ scan	*R*_int_ = 0.061
Absorption correction: multi-scan (*SCALA*; Evans, 2006)	θ_max_ = 38.1°, θ_min_ = 3.4°
*T*_min_ = 0.960, *T*_max_ = 0.969	*h* = −10→10
7090 measured reflections	*k* = −10→10
2104 independent reflections	*l* = −10→10

### (IIa) 5a(RS),6(RS),7(RS),9a(SR),9b(SR)-5-Oxo-1,2,3,5,5a,6,7,9b-octahydro-7,9a-epoxypyrrolo[2,1-a]isoindole-6-carboxylic acid Refinement

**Table:**

Refinement on *F*^2^	Secondary atom site location: difference Fourier map
Least-squares matrix: full	Hydrogen site location: mixed
*R*[*F*^2^ > 2σ(*F*^2^)] = 0.099	H-atom parameters constrained
*wR*(*F*^2^) = 0.240	*w* = 1/[σ^2^(*F*_o_^2^) + (0.06*P*)^2^] where *P* = (*F*_o_^2^ + 2*F*_c_^2^)/3
*S* = 0.93	(Δ/σ)_max_ < 0.001
2104 reflections	Δρ_max_ = 0.45 e Å^−^^3^
155 parameters	Δρ_min_ = −0.36 e Å^−^^3^
0 restraints	Extinction correction: SHELXL-2014/7 (Sheldrick, 2015), Fc^*^=kFc[1+0.001xFc^2^λ^3^/sin(2θ)]^-1/4^
Primary atom site location: difference Fourier map	Extinction coefficient: 0.138 (11)

### (IIa) 5a(RS),6(RS),7(RS),9a(SR),9b(SR)-5-Oxo-1,2,3,5,5a,6,7,9b-octahydro-7,9a-epoxypyrrolo[2,1-a]isoindole-6-carboxylic acid Special details

**Table:**

Geometry. All esds (except the esd in the dihedral angle between two l.s. planes) are estimated using the full covariance matrix. The cell esds are taken into account individually in the estimation of esds in distances, angles and torsion angles; correlations between esds in cell parameters are only used when they are defined by crystal symmetry. An approximate (isotropic) treatment of cell esds is used for estimating esds involving l.s. planes.

### (IIa) 5a(RS),6(RS),7(RS),9a(SR),9b(SR)-5-Oxo-1,2,3,5,5a,6,7,9b-octahydro-7,9a-epoxypyrrolo[2,1-a]isoindole-6-carboxylic acid Fractional atomic coordinates and isotropic or equivalent isotropic displacement parameters (Å^2^)

**Table:**

	*x*	*y*	*z*	*U*_iso_*/*U*_eq_	
C1	0.1779 (3)	0.0068 (2)	0.2264 (2)	0.0322 (5)	
H1A	0.0733	−0.0331	0.1171	0.039*	
H1B	0.2531	−0.0668	0.2374	0.039*	
C2	0.1215 (3)	0.0169 (2)	0.3765 (2)	0.0324 (5)	
H2A	0.0900	−0.0932	0.4081	0.039*	
H2B	0.0181	0.0592	0.3486	0.039*	
C3	0.2874 (2)	0.1392 (2)	0.5222 (2)	0.0288 (5)	
H3A	0.2529	0.2089	0.5934	0.035*	
H3B	0.3628	0.0797	0.5949	0.035*	
N4	0.37879 (19)	0.23864 (17)	0.42848 (16)	0.0261 (4)	
C5	0.5531 (2)	0.3199 (2)	0.4832 (2)	0.0250 (4)	
O5	0.65982 (17)	0.35299 (15)	0.63512 (13)	0.0299 (3)	
C5A	0.5950 (2)	0.3619 (2)	0.3297 (2)	0.0233 (4)	
H5A	0.5949	0.4769	0.3127	0.028*	
C6	0.7574 (2)	0.32415 (19)	0.3142 (2)	0.0245 (4)	
H6	0.8150	0.2754	0.4140	0.029*	
C7	0.6593 (3)	0.1818 (2)	0.1503 (2)	0.0276 (5)	
H7	0.7338	0.1138	0.1333	0.033*	
C8	0.5722 (2)	0.2548 (2)	−0.0027 (2)	0.0264 (5)	
H8	0.6108	0.2775	−0.0921	0.032*	
C9	0.4292 (3)	0.2805 (2)	0.0152 (2)	0.0284 (5)	
H9	0.3431	0.3223	−0.0598	0.034*	
C9A	0.4358 (2)	0.2276 (2)	0.1826 (2)	0.0256 (5)	
C9B	0.2840 (2)	0.1875 (2)	0.24142 (19)	0.0267 (5)	
H9B	0.2022	0.2541	0.1950	0.032*	
O10	0.51146 (16)	0.09341 (13)	0.18544 (14)	0.0258 (3)	
C11	0.8967 (2)	0.4657 (2)	0.2990 (2)	0.0259 (5)	
O11	0.88416 (17)	0.60281 (15)	0.27854 (15)	0.0338 (4)	
O12	1.04071 (16)	0.42233 (14)	0.31124 (15)	0.0312 (4)	
H12	1.1355	0.5093	0.3163	0.047*	

### (IIa) 5a(RS),6(RS),7(RS),9a(SR),9b(SR)-5-Oxo-1,2,3,5,5a,6,7,9b-octahydro-7,9a-epoxypyrrolo[2,1-a]isoindole-6-carboxylic acid Atomic displacement parameters (Å^2^)

**Table:**

	*U*^11^	*U*^22^	*U*^33^	*U*^12^	*U*^13^	*U*^23^
C1	0.0352 (10)	0.0244 (9)	0.0294 (8)	−0.0016 (8)	0.0132 (7)	−0.0032 (7)
C2	0.0364 (10)	0.0254 (9)	0.0311 (8)	−0.0003 (8)	0.0158 (7)	0.0030 (7)
C3	0.0365 (9)	0.0234 (9)	0.0290 (8)	0.0035 (8)	0.0201 (6)	0.0019 (7)
N4	0.0308 (8)	0.0214 (7)	0.0246 (6)	0.0012 (6)	0.0152 (5)	−0.0014 (5)
C5	0.0316 (9)	0.0159 (7)	0.0263 (8)	0.0045 (7)	0.0131 (6)	−0.0028 (6)
O5	0.0364 (7)	0.0281 (6)	0.0205 (5)	0.0017 (5)	0.0130 (4)	−0.0033 (5)
C5A	0.0284 (9)	0.0177 (8)	0.0227 (7)	0.0022 (7)	0.0131 (6)	−0.0005 (6)
C6	0.0355 (9)	0.0164 (8)	0.0232 (7)	0.0043 (7)	0.0165 (6)	0.0005 (6)
C7	0.0335 (9)	0.0192 (8)	0.0277 (8)	−0.0004 (7)	0.0169 (6)	−0.0048 (6)
C8	0.0373 (10)	0.0199 (8)	0.0209 (7)	0.0025 (7)	0.0158 (6)	−0.0026 (6)
C9	0.0368 (10)	0.0211 (8)	0.0229 (8)	0.0029 (8)	0.0118 (7)	−0.0001 (6)
C9A	0.0344 (9)	0.0168 (8)	0.0239 (7)	0.0043 (7)	0.0128 (6)	0.0000 (6)
C9B	0.0327 (9)	0.0214 (8)	0.0240 (8)	0.0032 (7)	0.0134 (6)	−0.0021 (6)
O10	0.0337 (6)	0.0164 (5)	0.0277 (5)	0.0033 (5)	0.0165 (4)	−0.0009 (4)
C11	0.0316 (9)	0.0227 (9)	0.0204 (7)	0.0024 (7)	0.0126 (6)	−0.0045 (6)
O11	0.0375 (7)	0.0228 (6)	0.0390 (6)	0.0042 (6)	0.0170 (5)	0.0031 (5)
O12	0.0309 (7)	0.0232 (6)	0.0403 (6)	0.0040 (5)	0.0189 (5)	0.0015 (5)

### (IIa) 5a(RS),6(RS),7(RS),9a(SR),9b(SR)-5-Oxo-1,2,3,5,5a,6,7,9b-octahydro-7,9a-epoxypyrrolo[2,1-a]isoindole-6-carboxylic acid Geometric parameters (Å, º)

**Table:**

C1—C2	1.532 (3)	C6—C11	1.500 (3)
C1—C9B	1.534 (3)	C6—C7	1.590 (2)
C1—H1A	0.9900	C6—H6	1.0000
C1—H1B	0.9900	C7—O10	1.427 (2)
C2—C3	1.552 (2)	C7—C8	1.521 (3)
C2—H2A	0.9900	C7—H7	1.0000
C2—H2B	0.9900	C8—C9	1.344 (3)
C3—N4	1.472 (2)	C8—H8	0.9500
C3—H3A	0.9900	C9—C9A	1.522 (2)
C3—H3B	0.9900	C9—H9	0.9500
N4—C5	1.341 (2)	C9A—O10	1.446 (2)
N4—C9B	1.485 (2)	C9A—C9B	1.513 (3)
C5—O5	1.250 (2)	C9B—H9B	1.0000
C5—C5A	1.526 (3)	C11—O11	1.218 (2)
C5A—C6	1.540 (3)	C11—O12	1.336 (2)
C5A—C9A	1.576 (2)	O12—H12	0.9239
C5A—H5A	1.0000		
			
C2—C1—C9B	102.15 (14)	C11—C6—H6	108.7
C2—C1—H1A	111.3	C5A—C6—H6	108.7
C9B—C1—H1A	111.3	C7—C6—H6	108.7
C2—C1—H1B	111.3	O10—C7—C8	102.71 (15)
C9B—C1—H1B	111.3	O10—C7—C6	99.48 (13)
H1A—C1—H1B	109.2	C8—C7—C6	109.15 (14)
C1—C2—C3	104.39 (16)	O10—C7—H7	114.6
C1—C2—H2A	110.9	C8—C7—H7	114.6
C3—C2—H2A	110.9	C6—C7—H7	114.6
C1—C2—H2B	110.9	C9—C8—C7	105.52 (17)
C3—C2—H2B	110.9	C9—C8—H8	127.2
H2A—C2—H2B	108.9	C7—C8—H8	127.2
N4—C3—C2	102.16 (13)	C8—C9—C9A	104.59 (17)
N4—C3—H3A	111.3	C8—C9—H9	127.7
C2—C3—H3A	111.3	C9A—C9—H9	127.7
N4—C3—H3B	111.3	O10—C9A—C9B	111.77 (14)
C2—C3—H3B	111.3	O10—C9A—C9	102.12 (14)
H3A—C3—H3B	109.2	C9B—C9A—C9	126.44 (16)
C5—N4—C3	127.10 (15)	O10—C9A—C5A	100.18 (14)
C5—N4—C9B	115.10 (15)	C9B—C9A—C5A	105.83 (13)
C3—N4—C9B	113.07 (13)	C9—C9A—C5A	107.50 (14)
O5—C5—N4	124.50 (18)	N4—C9B—C9A	102.11 (13)
O5—C5—C5A	127.21 (17)	N4—C9B—C1	101.06 (14)
N4—C5—C5A	108.28 (14)	C9A—C9B—C1	120.38 (16)
C5—C5A—C6	117.75 (15)	N4—C9B—H9B	110.7
C5—C5A—C9A	101.00 (14)	C9A—C9B—H9B	110.7
C6—C5A—C9A	101.86 (13)	C1—C9B—H9B	110.7
C5—C5A—H5A	111.7	C7—O10—C9A	96.02 (13)
C6—C5A—H5A	111.7	O11—C11—O12	123.30 (17)
C9A—C5A—H5A	111.7	O11—C11—C6	125.93 (18)
C11—C6—C5A	117.00 (15)	O12—C11—C6	110.77 (15)
C11—C6—C7	113.55 (15)	C11—O12—H12	113.8
C5A—C6—C7	99.72 (13)		
			
C9B—C1—C2—C3	−40.2 (2)	C6—C5A—C9A—O10	32.99 (16)
C1—C2—C3—N4	24.99 (19)	C5—C5A—C9A—C9B	27.56 (18)
C2—C3—N4—C5	−154.32 (18)	C6—C5A—C9A—C9B	149.26 (14)
C2—C3—N4—C9B	−0.1 (2)	C5—C5A—C9A—C9	165.01 (15)
C3—N4—C5—O5	−16.0 (3)	C6—C5A—C9A—C9	−73.29 (18)
C9B—N4—C5—O5	−169.80 (16)	C5—N4—C9B—C9A	8.4 (2)
C3—N4—C5—C5A	163.66 (15)	C3—N4—C9B—C9A	−149.09 (15)
C9B—N4—C5—C5A	9.9 (2)	C5—N4—C9B—C1	133.04 (17)
O5—C5—C5A—C6	47.1 (2)	C3—N4—C9B—C1	−24.4 (2)
N4—C5—C5A—C6	−132.62 (15)	O10—C9A—C9B—N4	85.85 (15)
O5—C5—C5A—C9A	156.89 (18)	C9—C9A—C9B—N4	−148.99 (15)
N4—C5—C5A—C9A	−22.81 (19)	C5A—C9A—C9B—N4	−22.27 (18)
C5—C5A—C6—C11	−123.93 (16)	O10—C9A—C9B—C1	−24.8 (2)
C9A—C5A—C6—C11	126.75 (15)	C9—C9A—C9B—C1	100.3 (2)
C5—C5A—C6—C7	113.23 (14)	C5A—C9A—C9B—C1	−132.96 (15)
C9A—C5A—C6—C7	3.91 (16)	C2—C1—C9B—N4	38.51 (18)
C11—C6—C7—O10	−165.65 (15)	C2—C1—C9B—C9A	149.76 (16)
C5A—C6—C7—O10	−40.40 (16)	C8—C7—O10—C9A	−49.46 (13)
C11—C6—C7—C8	−58.5 (2)	C6—C7—O10—C9A	62.79 (14)
C5A—C6—C7—C8	66.71 (18)	C9B—C9A—O10—C7	−171.68 (12)
O10—C7—C8—C9	30.75 (16)	C9—C9A—O10—C7	50.59 (14)
C6—C7—C8—C9	−74.15 (18)	C5A—C9A—O10—C7	−59.96 (14)
C7—C8—C9—C9A	1.83 (16)	C5A—C6—C11—O11	−8.9 (2)
C8—C9—C9A—O10	−33.38 (16)	C7—C6—C11—O11	106.5 (2)
C8—C9—C9A—C9B	−162.43 (15)	C5A—C6—C11—O12	171.02 (12)
C8—C9—C9A—C5A	71.53 (17)	C7—C6—C11—O12	−73.58 (19)
C5—C5A—C9A—O10	−88.71 (15)		

### (IIa) 5a(RS),6(RS),7(RS),9a(SR),9b(SR)-5-Oxo-1,2,3,5,5a,6,7,9b-octahydro-7,9a-epoxypyrrolo[2,1-a]isoindole-6-carboxylic acid Hydrogen-bond geometry (Å, º)

**Table:**

*D*—H···*A*	*D*—H	H···*A*	*D*···*A*	*D*—H···*A*
O12—H12···O5^i^	0.92	1.70	2.607 (2)	165
C9—H9···O11^ii^	0.95	2.42	3.362 (3)	172

Symmetry codes: (i) −*x*+2, −*y*+1, −*z*+1; (ii) −*x*+1, −*y*+1, −*z*.
